# Intermittent pneumatic compression devices for the prevention and treatment of breast cancer-related lymphedema—a systematic review and meta-analysis

**DOI:** 10.1007/s00520-025-10159-8

**Published:** 2025-11-22

**Authors:** Liyao Su, Haihong Huang, Yang Tong, Lu Dong, Chenxuan Gu, Siqi Zhuang, Shuyao Bai, Yongmei Jin

**Affiliations:** 1 Seventh People’s Hospital of Shanghai University of Traditional Chinese Medicine, Shanghai, China; 2 Kunshan Blood Center, Suzhou, China; 3 School of Nursing, Shanghai University of Traditional Chinese Medicine, Shanghai, China; 4 Chinese Medicine Yueyang Hospital of Integrated Traditional Chinese and Western Medicine, Shanghai University of Traditional, Shanghai, China

**Keywords:** Intermittent pneumatic compression devices, Breast cancer, Lymphedema, Prevention, Treatment

## Abstract

**Aims:**

This systematic review and meta-analysis aims to provide an up-to-date assessment of the efficacy of intermittent pneumatic compression (IPC) devices in preventing and treating breast cancer-related lymphedema (BCRL).

**Methods:**

From establishment to 2025–03-21, randomized controlled trials of IPC in the prevention and treatment of BCRL were searched and included in the databases of PubMed, Embase, Web of Science, The Cochrane Library, CNKI, WanFang Data Knowledge Service (WanFang), and SinoMed. Two researchers used inclusion and exclusion criteria to choose literature and assess its quality. RevMan 5.4 software was used for meta-analysis.

**Results:**

We found 14 randomized controlled studies with 1397 patients after conducting a thorough search across several databases. Meta-analysis showed that IPC significantly reduced the incidence of BCRL after breast cancer surgery (*P* < 0.01, RR = 0.36, 95%CI = [0.22, 0.58]) and improved the extension function of the affected limb (*P* = 0.02, SMD = 2.77, 95%CI = [0.41, 5.12]). Subgroup analyses indicated that lymphedema duration ≤ 24 months, IPC pressure ≤ 40 mmHg, treatment time > 2 weeks, and evaluation time ≤ 2 months were associated with better limb volume outcomes (all *P* < 0.05). However, no significant effects were observed on other joint movements or the patient’s subjective symptoms.

**Conclusion:**

IPC devices can effectively prevent the occurrence of BCRL, likely due to enhanced lymphatic return and reduced interstitial fluid accumulation, and early IPC intervention (≤ 40 mmHg pressure, > 2-week duration) is recommended for prevention, while combined therapies may be needed for established lymphedema. However, its limited therapeutic efficacy in chronic lymphedema may be related to irreversible structural damage in advanced cases. We need further rigorous, multicenter studies to optimize IPC protocols and clarify its role in BCRL management.

**Trial registration:**

PROSPERO has registered this study under the CRD42025631301.

**Supplementary information:**

The online version contains supplementary material available at 10.1007/s00520-025-10159-8.

## Introduction

An estimated 2.3 million cases of breast cancer were reported globally in 2023 [1]. It is the most frequent malignant neoplasm in women and poses a serious risk to their lives and health. Following axillary clearance and/or radiation therapy for breast cancer, secondary lymphedema is a common side effect [2]. The progression of BCRL is multifaceted, shaped by local and regional treatment approaches, individual lymphatic regeneration ability, and possibly alterable risk factors such as body mass index (BMI) [3–5]. Approximately 20% of women get upper limb lymphedema subsequent to breast cancer therapy, including axillary dissection [6]; breast cancer-related lymphedema diminishes the quality of life by substantially impacting the physical, mental, and emotional well-being of patients [7]. Consequently, optimizing postoperative recovery for breast cancer and effectively preventing and treating lymphedema are of paramount importance.

Complete Decongestive Therapy (CDT) is the usual treatment for individuals with BCRL [8], encompassing manual lymphatic drainage (MLD), multi-layer short stretch compression bandaging (CB), exercise, and diligent skin care of the affected region [9]. While it has yielded favorable outcomes in lymphedema management, it imposes stringent physical and temporal demands, particularly with MLD and CB [10]. It must be conducted by healthcare experts who are specially trained and certified as lymphedema therapists [11]. Lymphedema, an incurable yet manageable chronic and progressive condition, necessitates prolonged home care alongside extensive hospital therapy. Consequently, it is impractical for patients or their families to anticipate home treatment and prevention of BCRL by CDT.

Intermittent pneumatic compression (IPC) has been utilized for the treatment of BCRL since the 1950 s [3]; it is frequently employed in conjunction with CDT to augment the therapeutic efficacy [12]. IPC therapy is a specific device that uses an inflated sleeve to apply controlled, constant pressure to the afflicted limb, facilitating lymphatic drainage [13]. According to research, appropriate IPC use has rare side effects [14], making it a safer and more efficient treatment for lymphedema than invasive techniques. Simultaneously, it is a more uniform and straightforward treatment for patients, a therapeutic instrument for both inpatient and outpatient care, and part of the patient’s home care routine [15].

Both domestic and international researchers have examined the role of IPC in the prevention and treatment of BCRL; however, the superiority of IPC over conventional nursing methods remains contentious, as certain studies [16–20] have indicated that IPC plays a significant role in BCRL prevention. According to other researchers [21, 22], as compared to the intervention group, the effect of IPC on lowering lymphedema in the afflicted limb is not statistically significant. This needs to be confirmed by more research. There are a few systematic review publications regarding the effectiveness of IPC in the prevention or treatment of BCRL, according to a literature review. A comprehensive, methodical assessment of the relevant research data is crucial, especially in light of the continuous accumulation of knowledge in recent years. In order to conduct a meta-analysis on the effectiveness of IPC in the prevention and treatment of BCRL, this study gathered published studies up until September 30, 2024, to improve clinical nurses’ ability to provide the best possible treatment plan.

## Methods

### Search strategy

**Fig. 1:**
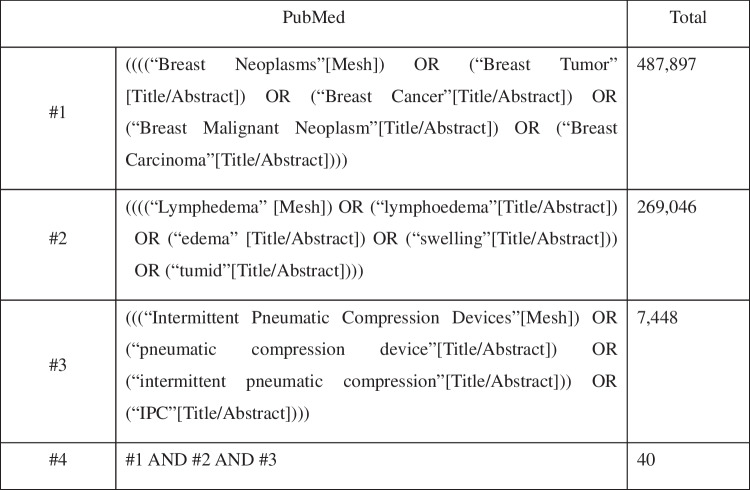
PubMed search strategy

Using the following terms: ([“Breast Neoplasms” OR “Breast Tumor” OR “Breast Cancer” OR “Breast Malignant Neoplasm” OR “Breast Carcinoma”] AND [“Lymphedema” OR “lymphoedema” OR “edema” OR “swelling” OR “tumid”] AND [“Intermittent Pneumatic Compression Devices” OR “pneumatic compression device” OR “intermittent pneumatic compression” OR “IPC”]). The computer system was queried in PubMed, Embase, Web of Science, The Cochrane Library, CNKI, Wan Fang, and SinoMed from inception to March 2015. The specific retrieval approach is illustrated in Fig. 1, using PubMed as a case study. Publication status is unrestricted, and the languages permitted are confined to English and Chinese. Grey literature sources, including ClinicalTrials.gov, WHO ICTRP, OpenGrey, ProQuest Dissertations & Theses, and major conference abstracts (ASCO, ISL), were searched using the same strategy.

### Eligibility criteria

The references selected were randomized controlled trials (RCTs) that compared treatment and prevention using IPC devices or IPC devices in conjunction with other measures against traditional methods. The criteria for inclusion and exclusion were as follows: (1) Study subjects (Patients, P): individuals aged 18 years or older undergoing surgical intervention for breast cancer; (2) Intervention (I): Patients in the intervention group received treatment with IPC devices or IPC devices in conjunction with additional interventions to address and prevent BCRL; (3) Control measures (C): Patients in the control group received standard nursing interventions; (4) Outcomes (O): The principal outcome measure of the IPC treatment’s efficacy was the variation in arm circumference pre- and post-treatment of the affected limb lymphedema, whereas the secondary outcome pertained to the patients’ subjective symptoms (such as limb pain, tightness, heaviness, numbness, etc.). The principal outcome measure employed to investigate the preventative effect of IPC was the incidence of lymphedema in the affected limb. The secondary indicator is the operational condition of the affected limb; (5) Study design (S): RCTs utilizing IPC devices for the prevention or treatment of BCRL, published in Chinese or English. Case series and review papers, duplicate publications, inaccurate data, and inadequate literature were omitted.

### Selection procedure

Two researchers separately conducted literature screening and data extraction according to the defined criteria, while cross-validation was performed to verify correctness. The title and abstract were reviewed for the initial screening during the literature search, followed by a full-text evaluation to discard publications that did not satisfy the inclusion criteria. Disagreements were resolved by discussion or by consultation with a third reviewer.

### Data acquisition

The data gathering was performed independently by two researchers and compiled into a Microsoft Excel spreadsheet. We addressed discrepancies in data extraction by engaging in dialogue and, when necessary, reaching out to a third reviewer. Recorded baseline participant characteristics encompassed age, clinical stage of breast cancer, history of radiation, presence of comorbidities, educational attainment, marital status, and pre-treatment circumference of the afflicted limb. The period of follow-up was not a restrictive criterion for research inclusion. Appendix 1 presents comprehensive inclusion and exclusion criteria for each study.

### Quality evaluation for bias risk

The quality evaluation of the included randomized controlled trials (RCTs) was performed utilizing the Cochrane Risk of Bias Tool (2.0), which assesses studies across five domains: randomization process, deviations from intended interventions, missing outcome data, outcome measurement, and selection of reported results. Each domain was classified as “Yes,” “Probably Yes,” “No,” “Probably No,” or “No Information.” The studies were classified as “low risk,” “some concerns,” or “high risk,” facilitating a thorough assessment of the objectivity and precision of the published results.

### Data analysis

A meta-analysis was performed with RevMan 5.4. Continuous data were evaluated using mean difference (MD) or standardized mean difference (SMD). When only pre- and post-treatment data were available within the same intervention group, effect sizes were calculated as standardized mean change using Hedges’g with small-sample correction, assuming a correlation coefficient (*r* = 0.5) between baseline and post-treatment values.

Conversely, binary data were evaluated using relative risk (RR), with 95% confidence intervals (CI) provided for all effect sizes. Heterogeneity was evaluated utilizing *I*^2^ and *Q* tests. A fixed-effect model was utilized when *I*^2^ < 50% and *P* ≥ 0.1; a random-effects model was applied for meta-analysis when *I*^2^ > 50%, with subgroup analyses stratified by lymphedema duration, IPC pressure, and treatment time to address heterogeneity. Funnel plots and Egger’s test were employed to evaluate publication bias, while sensitivity analysis was performed using Stata 18.0 to verify the robustness of the findings. Statistical significance was established at *P* < 0.05. The certainty of evidence for each main outcome was evaluated using the GRADE approach, considering study limitations, inconsistency, indirectness, imprecision, and publication bias.

**Table:** 

PubMed		Total
#1	((((“Breast Neoplasms”[Mesh]) OR (“Breast Tumor” [Title/Abstract]) OR (“Breast Cancer”[Title/Abstract]) OR (“Breast Malignant Neoplasm”[Title/Abstract]) OR (“Breast Carcinoma”[Title/Abstract])))	487,897
#2	((((“Lymphedema” [Mesh]) OR (“lymphoedema”[Title/Abstract]) OR (“edema” [Title/Abstract]) OR (“swelling”[Title/Abstract])) OR (“tumid”[Title/Abstract])))	269,046
#3	(((“Intermittent Pneumatic Compression Devices”[Mesh]) OR (“pneumatic compression device”[Title/Abstract]) OR (“intermittent pneumatic compression”[Title/Abstract])) OR (“IPC”[Title/Abstract])))	7448
#4	#1 AND #2 AND #3	40

## Results

### Selection of studies

**Fig. 2:**
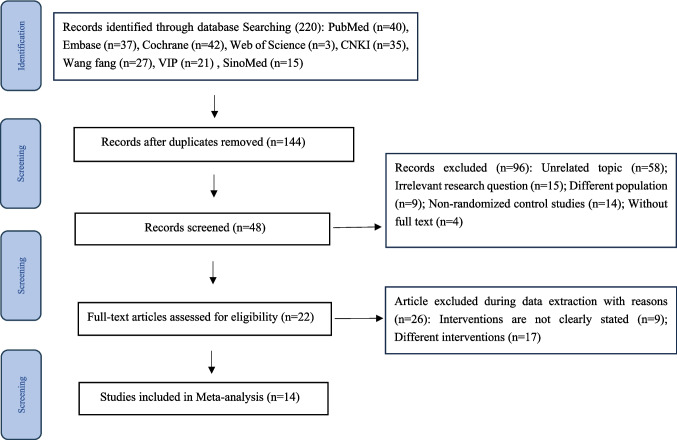
PRISMA flow diagram

The research selection procedure is defined in the PRISMA flowchart (Fig. 2). Initially, 48 publications (26 in English and 22 in Chinese) were selected by title and abstract screening. A total of 22 papers were examined, leading to the inclusion of 14 articles (seven [21–27] in English and seven [16–20, 28, 29] in Chinese) that satisfied the established criteria. The research comprised nine articles [21–29] addressing the therapeutic effect of IPC and five articles [16–20] focusing on its preventive effects. No unpublished or grey literature studies met the inclusion criteria, and thus none were included in the final analysis. Comprehensive criteria for each study are described in Appendix 1. According to the Cochrane assessment, the certainty of evidence ranged from low to moderate across outcomes, reflecting limitations related to risk of bias, heterogeneity, and small sample sizes in included trials. The details of the included studies are shown in Table 1.

**Table 1:** Characteristics of randomized controlled trails included in the systematic review

Study	Date published	Cases (T/C)	Age (T/C)	Specific intervention measures	Specific control measures	Method of evaluate outcome	Evaluation time
Uzkeser et al. [26]	2015	16/15	N/A	CDT + IPC: The IPC pressure is 40 mmHg, the treatment time is 45 min, and the model number (MK400) is five times/week for 3 weeks	CDT	The volume measurement method was used to compare before and after treatment. The skin thickness was measured by ultrasound with a Toshiba Xarioprime 7.5 MHz probe. Patients were asked about their pain complaints and rated using a Visual Analog Scale (VAS)	3 weeks
Sanal-Toprak et al. [27]	2019	22/24	N/A	IPC + compressive bandages + home exercise program: The IPC pressure is 50 ~ 80 mmHg, 30 min/time, the inflation interval is 60 s, three times/week, treatment continued for 5 weeks	MLD + compressive bandages + home exercise program	The inelastic tape measure was used to measure the circumference difference of both arms to determine the degree of edema. The VAS was used to evaluate shoulder pain, tightness, and heaviness and to measure shoulder range of motion ROM	5 weeks
Gurdal et al. [21]	2012	15/15	50.13 ± 10.83/58.13 ± 10.54	MLD + IPC: The IPC pressure is 25 mmHg, 45 min/time, and MLD 15 min/time, once/day; treatment was performed on alternate days for 6 weeks	MLD pressure is 30 mmHg, 30 min/time, and was treated every other day for 6 weeks	Used specific formulas to calculate the limb volumes from the measurements of limb circumferences. EORTC-QLQ and ASES tests were applied to each patient in both groups	6 weeks
Haghighat et al. [25]	2010	56/56	52.7 ± 10.8/53.4 ± 11.4	CDT + IPC: The IPC pressure is 40 mmHg, 30 min/time, 5 days/week; MLD 15 ~ 20 min/time	CDT (MLD is 45 min, 5 days/week)	The volume of edema was measured using water displacement method with a subjective symptom questionnaire	3 months
Tastaban et al. [22]	2020	38/38	N/A	CDT + IPC: The IPC pressure is 30 ~ 40 mmHg, 60–90 min/day, 5 days/week for 4 weeks	CDT	The truncated cone formula calculates the volume of each limb; an elastic non-stretch tape was measured to measure the circumference. A Visual Analog Scale (VAS) and a numerical rating scale of 0–10 were used to evaluate the clinical symptoms of pain, heaviness, and tightness of the affected arm	4 weeks
Szolnoky et al. [24]	2009	14/13	N/A	CDT + IPC: The IPC pressure is 40 ~ 50 mmHg, 30 min/time; MLD 30 min/time, once/day, a total of 10 days	CDT (MLD is 60 min, 10 days)	Expert diagnosis, circumference measurement, subjective symptom questionnaire	3 months
Szuba et al. [23]	2002	12/11	68.8 ± 9.11/65 ± 10.8	CDT + IPC: The IPC pressure is 40 ~ 50 mmHg, 30 min/time, 10 days	CDT(MLD is 30 to 60 min, 10 days)	Hydrometry: measuring limb volume; tissue tension measures skin elasticity; angle joint mobility is measured by degree measurement	40 days
YuNan, H et al. [29]	2021	39/39	35.46 ± 6.28/36.28 ± 7.56	Routine care + IPC: The IPC pressure is 7 ~ 10 kPa, each treatment was 20 min, twice a day, and 10 d was a course of treatment, a total of two courses of treatment	Routine care (functional exercise、limb massage)	The circumference of the affected limb before and after treatment was measured and compared with that of the healthy side and before treatment to judge the treatment of edema. The good and good rate of rehabilitation was judged by the motion degree of the affected limb. The time required for the recovery of the affected limb and the length of hospital stay were counted	Two courses
YongHong,L et al. [28]	2012	30/28	49.5 ± 5.8/52.0 ± 4.0	Routine care + IPC: The IPC pressure is 7 ~ 10 kPa, 20 min/time, two times/day, 10 days for a course, a total of two courses of treatment	Routine care	The circumference of the swelling area and the corresponding joint motion of the affected limb were measured and compared	3 weeks
Yi, C et al. [19]	2021	43/43	46.35 ± 6.27/47.82 ± 5.79	Routine care + IPC: The IPC pressure is 60 ~ 120 mmHg, 20 min/time, two times/day, 10 days for a course, a total of three courses of treatment	Routine care (rehabilitation guidance, functional exercise)	The grade of lymphedema of the affected limb was determined by circumferential diameter measurement. The functional recovery of the affected limb was evaluated according to ROM joint motion criteria	Three courses
Na, L et al. [20]	2021	43/42	52.8 ± 3.2/52.5 ± 3.8	Routine care + IPC: An air wave pressure therapy instrument was used from 7 days after surgery, the pressure range was set to 60 ~ 120 mmHg, each treatment was 20 min, twice a day, and 10 d was a course of treatment, a total of three courses of treatment	Routine care (functional exercise)	The circumference of the affected limb was measured with a soft tape measure to determine the degree of lymphedema. A protractor was used to measure joint motion. Application of the Breast Cancer Quality of Life Scale (FACT-B)	Three courses
YuPing,W et al. [16]	2015	50/50	N/A	Routine care + IPC: Starting from the third day after surgery, the pressure was set at 7 ~ 10 kPa, 30 min/time, twice/day, 10 d was a course of treatment, and two courses were performed	Routine care (rehabilitation guidance, functional exercise)	The anxiety self-assessment form (SAS) was used to judge the psychological status of patients. Circumference of different parts of the affected limb was measured with a tape measure	Two courses
Jie,P et al. [17]	2016	284/283	N/A	Routine care + IPC: Air wave pressure therapy instrument (DL2003V3) was used from 7 days after surgery, the pressure range was set to 60 ~ 120 mmHg, each treatment was 20 min, twice a day, and 10 d was a course of treatment, a total of three courses of treatment	Routine care (psychological counseling, dietary guidance, daily routine care, functional exercise)	A special protractor was used to measure the motion of the shoulder joint on the affected side to evaluate the functional status of the affected limb. Use a tape measure to measure the circumference of the affected limb to determine the degree of swelling	Three courses
PinKun,L et al. [18]	2019	39/39	66.12 ± 5.84/67.78 ± 5.64	Routine care + IPC: The IPC pressure is 20 ~ 130 mmHg, 25 min ~ 30 min/time, the inflation interval is 20 s, two times/day, 10 days for a course	Routine care (health education, physical exercise, psychological care)	The diameter of affected limb was measured by tape measure before and after treatment	N/A

### Participants

Fourteen articles involving 1397 patients were examined, comprising 701 in the experimental group and 696 in the control group. The age of patients was inconsistently documented. Among eight [18–21, 23, 25, 28, 29] studies, the youngest cohort was recorded by YuNan, H et al. [29] (experimental group: 35.46 ± 6.28, control group: 36.28 ± 7.56), while the oldest cohort was noted in Szuba et al. [23] (experimental group: 68.8 ± 9.11, control group: 65 ± 10.8). The average age in previous research varied from the mid-30 s to the mid-60 s. All patients were female and diagnosed pathologically with breast cancer. Comprehensive reporting encompassed lymphedema length in six studies [22–27], BMI and postoperative treatment history in five studies [21, 22, 25–27], and surgical lymph node dissection in four studies [22, 25–27]. The baseline statistics were consistent and comparable across the groups.

### Results of the study bias risk assessment were included

We assessed the bias risk of the included studies using the Cochrane Risk of Bias Tool (2.0). Three studies [17, 21, 22] utilized a computer-generated random sequence for randomization, whereas three [18, 20, 29] employed a random number table. A study [26] randomized patients continuously and alternately according to their admission time. In research with insufficient randomization techniques, the risk of bias was assessed as “some concerns.” The outcome measurement of one article may exhibit inadvertent bias. Nevertheless, in the real instrument, aside from the blind intervention conducted only by the researcher, other evaluation outcomes may be influenced by the absence of blinding, which is insufficient to impact the assessment of the intervention’s efficacy. Consequently, the measurement bias is characterized as “some concerns.” Overall, eight articles [16, 17, 19, 23–25, 27, 28] were classed as “some concerns”, and six [18, 20–22, 26, 29] as “low risk”. Bias risk diagrams for the studies considered are displayed in Appendix 2, 3, and 4.

### Therapeutic efficacy of IPC on BCRL

Nine studies [21–29] documented the therapeutic effect of IPC on BCRL, of which six studies [21–26] employed CDT or a combination of CDT and IPC as intervention measures, while two [28, 29] utilized conventional therapy with IPC as intervention measures. A study [27] employed the compression bandage technique and a home workout regimen in conjunction with IPC as intervention strategies. The control group consisted of the compressed bandage approach and a home exercise regimen in conjunction with MLD.

#### Effect on limb volume

Limb volume alterations were employed as a diagnostic criterion for evaluating the extent of limb edema in five studies [21–25]. Of these, two studies [21, 22] exclusively documented the mean percentage of volume reduction pre- and post-treatment. Due to the inability to transform these data into a standardized format, they were omitted from the meta-analysis. Specifically, Gurdal et al. [21] reported a mean percentage reduction of 14.97% in the control group and 12.26% in the experimental group. The findings indicated that, in comparison to other interventions combined with CDT, CDT combined with IPC did not show a therapeutic advantage in reducing limb edema volume and even exhibited a lesser effect than the control group. Engin Tastaban et al. [22] reported a volume reduction of 45.58% in the control group and 50.07% in the intervention group. The disparity between the groups was not statistically significant.

**Fig. 3:**
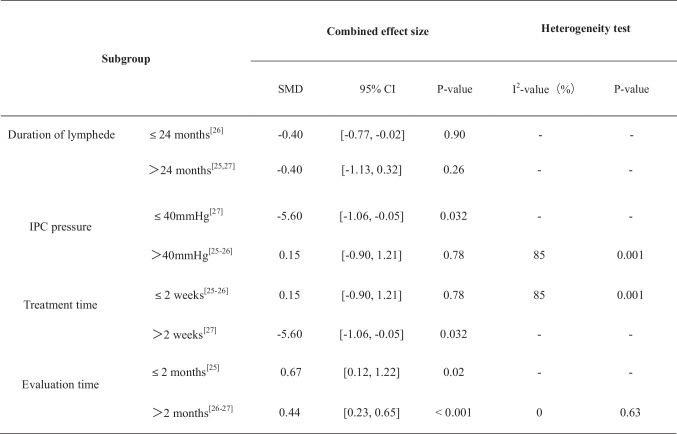
Subgroup analysis of IPC parameters on limb volume reduction

The remaining three studies [23–25] employed analogous measurement methods, allowing for their integration in the analysis. The results indicated statistical heterogeneity among the three studies (*P* = 0.001, *I*^2^ = 85%), which might be attributed to variations in lymphedema treatment protocols, such as the specific intervention techniques, IPC pressure settings, treatment durations, and timing of outcome measurements. Therefore, a random-effects model was applied. The pooled analysis showed no statistically significant difference between groups (SMD = 0.15, 95% CI = [− 0.90, 1.21], *P* = 0.78), indicating that IPC did not produce a consistent additional benefit in reducing limb volume overall. Subgroup analysis revealed that lymphedema duration ≤ 24 months (SMD =  − 0.40, 95% CI = [− 0.77, − 0.02]), IPC pressure ≤ 40 mmHg (SMD =  − 5.60, 95% CI = [− 1.06, − 0.05]), treatment time > 2 weeks (SMD =  − 5.60, 95% CI = [− 1.06, − 0.05]), and evaluation period ≤ 2 months (SMD = 0.67, 95% CI = [0.12, 1.22]) demonstrated statistically significant improvements (*P* < 0.05). Conversely, subgroup results for lymphedema duration > 24 months, IPC pressure > 40 mmHg, and evaluation period > 2 months were not significant (*P* > 0.05). Figure 3 presents the detailed outcomes of the subgroup analysis.

**Table:** 

Subgroup		Combined effect size			Heterogeneity test	
		SMD	95% CI	*P* value	*I*^2^ value (%)	*P* value
Duration of lymphedema	≤ 24 months [25]	− 0.40	[− 0.77, − 0.02]	0.90	-	-
	> 24 months [24, 26]	− 0.40	[− 1.13, 0.32]	0.26	-	-
IPC pressure	≤ 40 mmHg [26]	− 5.60	[− 1.06, − 0.05]	0.032	-	-
	> 40 mmHg [24, 25]	0.15	[− 0.90, 1.21]	0.78	85	0.001
Treatment time	≤ 2 weeks [24, 25]	0.15	[− 0.90, 1.21]	0.78	85	0.001
	> 2 weeks [26]	− 5.60	[− 1.06, − 0.05]	0.032	-	-
Evaluation time	≤ 2 months [24]	0.67	[0.12, 1.22]	0.02	-	-
	> 2 months [25, 26]	0.44	[0.23, 0.65]	< 0.001	0	0.63

*SMD (standardized mean difference) is a measure used to quantify the difference between two groups in terms of the mean values of a particular outcome. It is standardized by dividing the mean difference by the pooled standard deviation, allowing for comparisons across different studies, even if they use different measurement scales. The 95% CI (95% confidence interval) provides a range of values within which the true effect size (SMD) is likely to fall with 95% confidence. It gives an indication of the precision of the estimate. The *P* value is a measure of the probability that the observed difference between the groups could have occurred by chance. It is used to determine statistical significance. A *P* value < 0.05 is typically considered statistically significant, indicating that the observed difference is unlikely to be due to chance. A *P* value ≥ 0.05 indicates that the observed difference is not statistically significant

#### Effect on limb circumference

Two studies [26, 27] assessed the effect of IPC on limb circumference. Uzkeser et al. [26] observed that following a 7-week intervention, the diameter of the afflicted limb in the IPC group considerably diminished (*P* < 0.05). Conversely, only the wrist circumference in the control group exhibited a statistically significant reduction (*P* < 0.05). This finding does not indicate any supplementary advantage of IPC for lymphedema management. Sanal-Toprak et al. [27] observed that at 5 weeks and 3 months post-intervention, the circumferences of the wrist, metacarpophalangeal joints, and medial epicondyle in both groups were significantly diminished compared to baseline (*P* < 0.05), although no significant intergroup differences were identified. The alterations in the five assessed levels of upper arm circumference were more significant at 3 months compared to 5 weeks in both groups.

#### Effect on subjective symptoms

Five studies [21, 22, 24–26] assessed subjective symptom outcomes. The investigations conducted by Uzkeser et al. [26], Gurdal et al. [21], Haghighat et al. [25], and Szolnoky et al. [24] revealed no statistically significant differences in pain, heaviness, paresthesia, or other symptoms in the affected limb of patients receiving IPC in conjunction with CDT when compared to the control group (*P* > 0.05). In contrast, Tastaban et al. [22] found that weight and tightness in the treated limbs were considerably reduced in the IPC + CDT group compared to controls (*P* < 0.05), whereas no statistically significant differences were observed for other subjective symptoms, including pain and depression (*P* > 0.05).

#### Influence on therapeutic efficacy

**Fig. 4:**
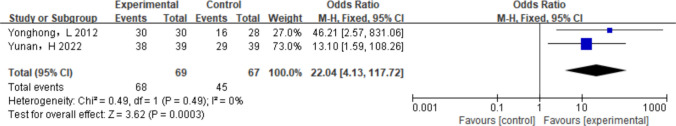
Meta-analysis of patient’s therapeutic effect after IPC treatment

Two studies [28, 29] assessed the treatment efficacy of IPC on lymphedema, classifying outcomes as “obvious,” “effective,” and “ineffective.” He et al. [29] evaluated therapy success by measuring the decrease in circumference diameter of the affected limb, whereas Luo et al. [28] concentrated on patient-reported symptoms, limb edema, and the recovery of joint function. A meta-analysis has shown that the experimental group achieved markedly better treatment than the control group (*P* < 0.01, RR = 22.04, 95% CI = [4.13, 117.72]). The pooled results suggested a possible trend toward better outcomes in the IPC group; however, considerable heterogeneity and small sample sizes limit the robustness of this finding. Thus, the therapeutic efficacy of IPC should be interpreted with caution. The results are depicted in Fig. 4.

#### Analysis of the sensitivity of IPC therapy on the effects of BCRL

**Fig. 5:**
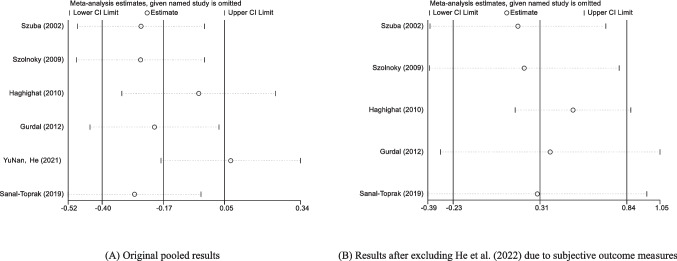
Sensitivity analysis of IPC therapeutic efficacy. *Fixed-effect model (*I*^2^ = 0%) in **A**; random-effects model (*I*^2^ = 45%) in **B**. Diamonds represent pooled effect sizes. Subjective symptom assessments in He et al. [29] may introduce bias.

The criteria for evaluating primary outcomes in the selected research are variable. Incorporating a standardized sensitivity analysis chart allows for the visual comparison and assessment of the impact of different outcome indicators on model robustness. Studies that use non-convertible data evaluate their potential influence on outcomes without including them in the analysis. Figure 5a indicates that the sensitivity analysis results for six studies [21, 23–25, 27, 29] are inconsistent. Upon removing the study by Yunan, He et al. [29], the aggregated results of the remaining studies attain statistical significance (95% CI = [− 0.23–0.84]), as illustrated in Fig. 5b. This implies that the outcome indicators in Yunan, He et al. [29] may possess a more significant subjective element, thus compromising the data’s accuracy.

#### Analysis of bias in IPC treatment on BCRL outcomes

**Fig. 6:**
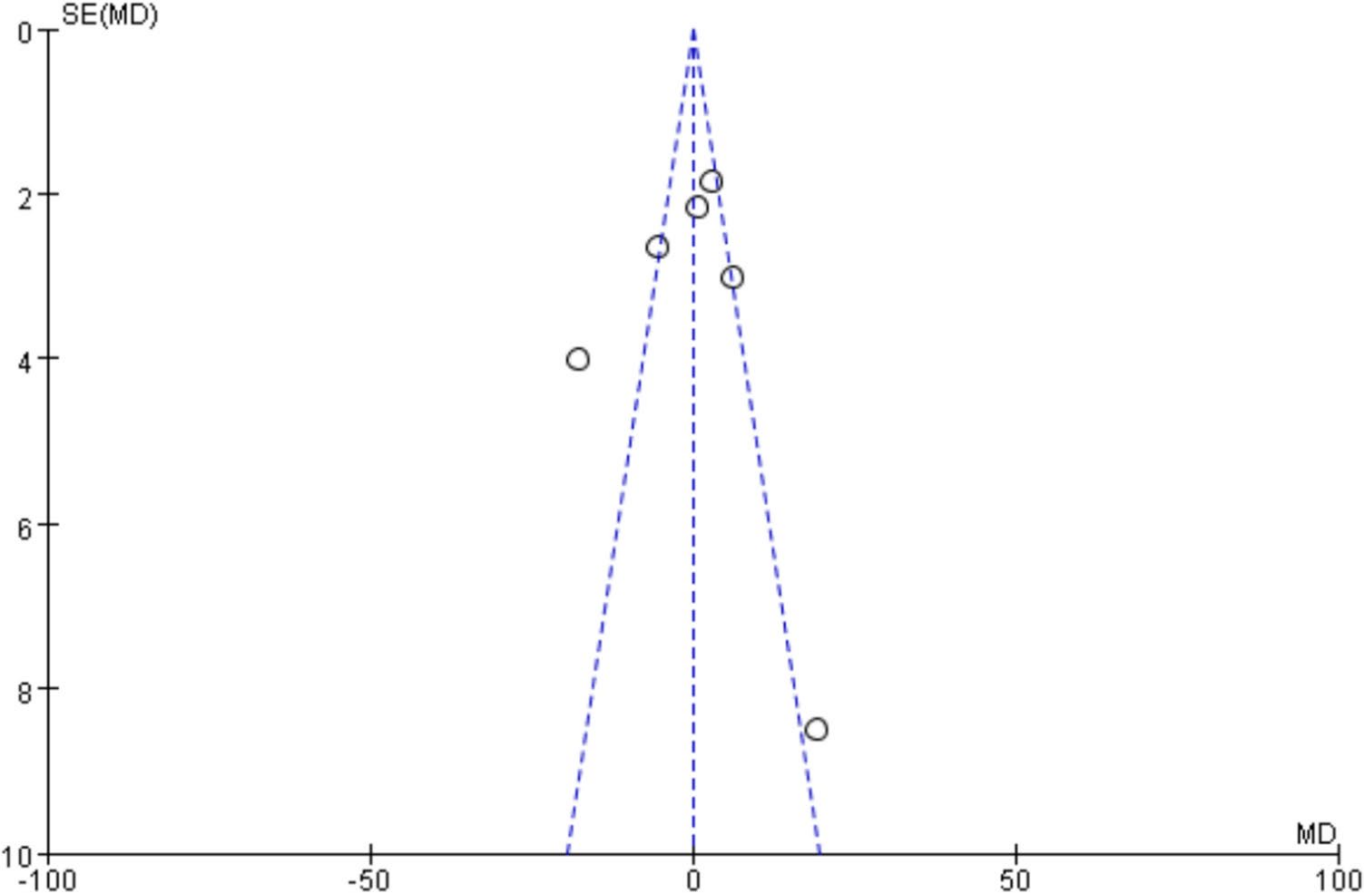
The funnel plot of studies in IPC treatment to BCRL effect

Egger’s test and the funnel plot were utilized to evaluate publication bias. The funnel plot (Fig. 6) exhibits a predominantly symmetric scatter distribution, indicating no publishing bias. The Egger test result (*P* > 0.05) further substantiates the lack of significant publication bias in this study.

### IPC’s preventive effect on BCRL

The impact of IPC on the prevention of lymphedema associated with breast cancer was investigated in five papers [16–20], all of which used IPC as an intervention in addition to standard treatment.

#### Prevalence of lymphedema

**Fig. 7:**
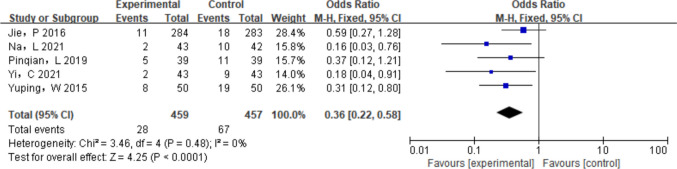
Meta-analysis of the incidence of lymphedema after breast cancer surgery between experimental group and the control group

The prophylactic effect of IPC on BCRL was evaluated in five studies [16–20], and the results showed no statistical heterogeneity (*P* = 0.48, *I*^2^ = 0%). A fixed-effect model was therefore applied. As shown in Fig. 7, the study found that the incidence of BCRL was significantly lower in the IPC group compared to the control group (*P* < 0.01, RR = 0.36, 95% CI = [0.22, 0.58]).

#### Effect on limb function

**Fig. 8:**
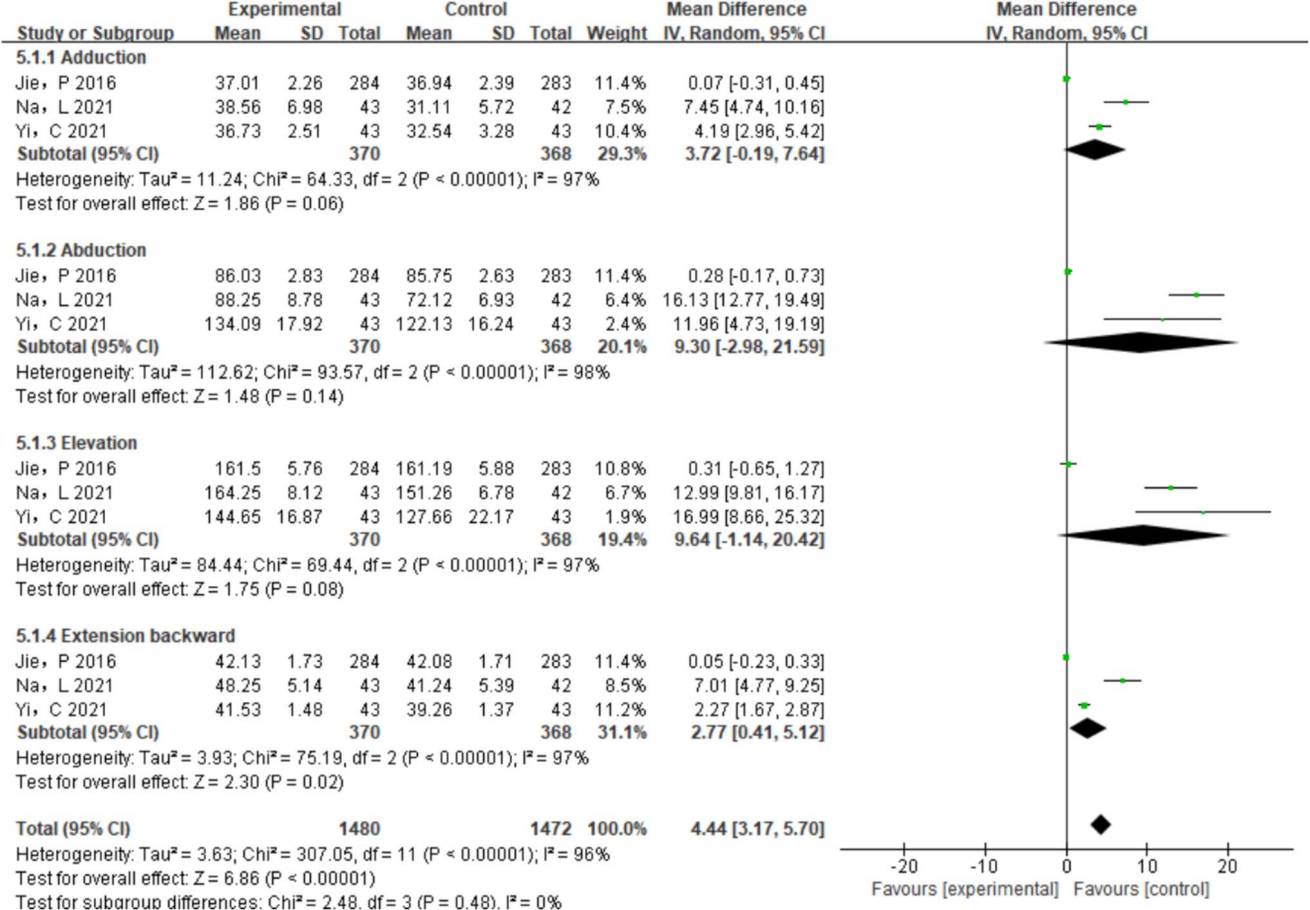
A subgroup analysis of IPC treatment for BCRL

The effect of IPC treatment on joint range of motion was assessed in three studies [17, 19, 20], which showed significant variability (*I*^2^ > 50%). The use of a random effects model for meta-analysis (Fig. 8) was required because of this variability, which most likely resulted from variations in specific therapy and the timing of outcome evaluations.

- Adduction function: IPC had no statistically significant effect of IPC on adduction function according to a meta-analysis of three trials [17, 19, 20] (*P* = 0.06, SMD = 3.72, 95% CI = [− 0.19, 7.64]).
- Abduction function: Likewise, no statistically significant results were seen for the abduction function (*P* = 0.14, SMD = 9.30, 95% CI = [− 2.98, 21.59]).
- Elevation: Elevation was not substantially impacted by IPC treatment (*P* = 0.08, SMD = 9.64, 95% CI = [− 1.14, 20.42]).
- Extension function: In contrast, IPC treatment considerably enhanced the afflicted limb’s extension function (*P* = 0.02, SMD = 2.77, 95% CI = [0.41, 5.12]). However, given the lack of biological plausibility and potential measurement variability, this result should be interpreted with caution.

#### Sensitivity analysis of IPC treatment intervention in BCRL

**Fig. 9:**
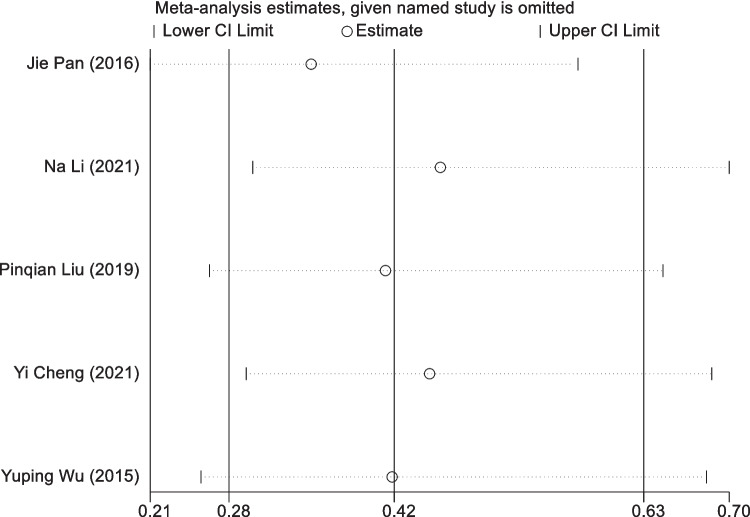
The sensitivity analysis results of studies in IPC treatment intervention in BCRL

A one-by-one exclusion procedure was used to do a sensitivity analysis on five experiments. The results demonstrated that the aggregated results of the subsequent trials were statistically significant (95% CI = [0.28, 0.63]), which was consistent with the original combined results (RR = 0.36, 95% CI = [0.22, 0.58]). This consistency indicates consistent outcomes, as shown in Fig. 9.

#### Analysis of bias in IPC treatment intervention for BCRL

**Fig. 10:**
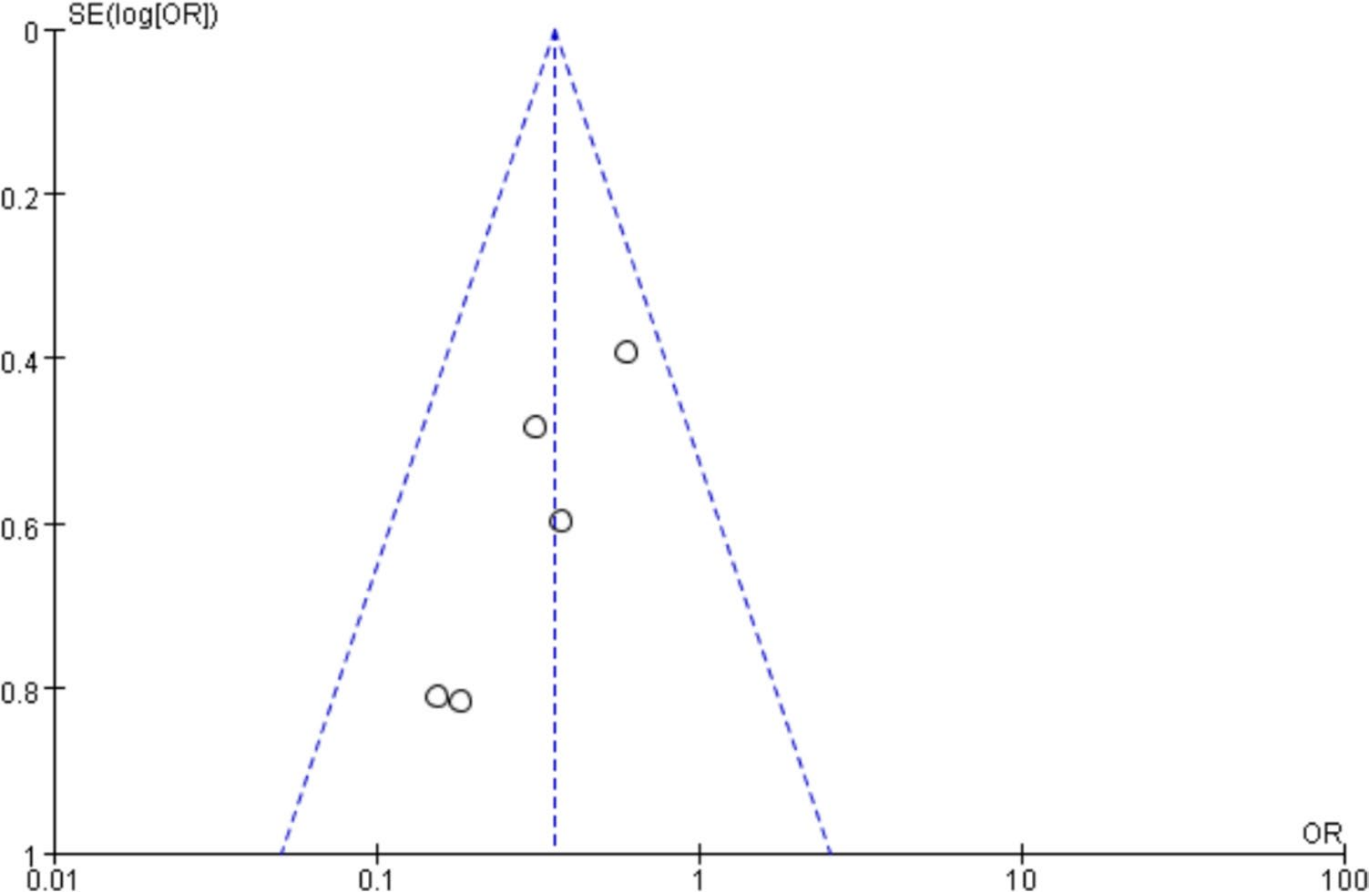
The funnel plot of studies in IPC treatment intervention in BCRL

To assess publication bias, the funnel plot and Egger’s test were employed. The funnel plot (Fig. 10) revealed a symmetric scatter distribution, indicating virtually no publishing bias. The Egger test, which yielded a *P* value of 0.058 (*P* > 0.05), confirmed this conclusion by showing no observable publication bias.

## Discussion

### Incidence of BCRL and the significance of physical therapy and IPC

About 20% of breast cancer patients experience BCRL after therapy, making it a common consequence [30]. Physical therapy is crucial for the treatment of BCRL because of its conservative and non-invasive approach [31]. After breast cancer surgery, IPC, a crucial physical therapy approach, is especially helpful in treating lymphedema.

For breast cancer-related lymphedema (BCRL), two conventional treatments are manual lymphatic drainage (MLD) and multilayer compression bandages (CB). The complexity of these procedures, the requirement for specialized personnel, and the possibility of chronic skin problems are some of its disadvantages [32, 33]. However, intermittent pneumatic compression (IPC) offers several advantages as a mechanical therapeutic tool. Because of its ease of use and ability to be used at home, patients are less dependent on certified lymphedema therapists [34]. Its portability also facilitates long-term adherence, which enhances the effectiveness and continuity of therapy [35]. Additionally, the action of the muscle pump is effectively replicated by IPC’s dynamic intermittent compression, promoting lymphatic return with less skin irritation and greater safety [35].

IPC can be used on its own for patients with mild lymphedema and as a routine postoperative treatment [36, 37]. It has shown exceptional efficacy in clinical settings in avoiding post-breast cancer surgery lymphedema. However, when combined with Complex Decongestive Therapy (CDT), IPC offers even more significant advantages, especially in improving limb function and reducing edema [38]. IPC can also be used in conjunction with other CDT therapies, including manual lymphatic drainage and functional exercises, to create a personalized treatment plan for each patient’s particular ailment and attain the best possible therapeutic result [10].

### Preventive efficacy and ideal duration of IPC

This study’s comprehensive analysis demonstrates that early IPC treatment can considerably lessen the risk of skin infections and limb dysfunction, improve patients’ postoperative quality of life, and reduce the incidence of postoperative lymphedema [16–20, 39]. While extending the treatment period to 12 weeks did not improve results, research by Pan et al. indicated that 5 weeks of IPC treatment can considerably reduce the incidence of lymphedema, indicating that the duration of IPC intervention has to be further optimized [17]. Although their participants were not restricted to patients with postoperative breast cancer lymphedema, a study by Ridner et al. likewise showed that the best course of treatment for cancer-related lymphedema is 1 month [39]. To develop a more accurate intervention timing model and provide a scientific foundation for clinical practice, future research should integrate existing data, conduct cohort studies with varying time gradients, and carry out long-term follow-up [40]. This is because postoperative recovery following breast cancer surgery is complicated.

IPC has proven to be quite effective in avoiding lymphedema associated with breast cancer. IPC has proven to be beneficial in preventing breast cancer-related lymphedema by simulating the physiological action of the muscle pump. Through gradual inflation and deflation, IPC promotes lymphatic and venous return, reduces interstitial fluid accumulation, and thereby decreases the risk of postoperative lymphedema [41, 42].

### Comparative efficacy of IPC in treatment of BCRL

When it comes to treating BCRL, IPC may be beneficial in improving lymphatic return and alleviating upper limb discomfort by promoting local circulation and reducing interstitial fluid accumulation. While these effects may help slow disease progression, the therapeutic impact appears modest and varies across studies. In addition to greatly easing the patient’s upper limb pain, the lowering of inflammation also delays the advancement of lymphedema. Additionally, long-term accumulation of lymphatic fluid may lead to progressive tissue fibrosis and lymphatic dysfunction, which could partly explain the limited therapeutic response observed in chronic lymphedema. However, this remains a theoretical explanation, as the included studies did not provide direct data confirming this mechanism. Future studies with histopathological or imaging assessments are needed to verify this hypothesis.

#### The extension function of the affected limb

This study observed an apparent improvement in the extension function of the affected limb; however, the underlying mechanism remains unclear, and this finding may be attributable to measurement variability or chance rather than a definitive therapeutic effect. Therefore, it should be interpreted with caution. However, as compared to the control group, there is no discernible difference in the treated limb’s other motor functions after the intervention. The rationale may be that distinct limb functional movements depend on diverse muscle groups, joint anatomy, and brain control mechanisms [43]. Consequently, acknowledging the constraints of monotherapy in managing breast cancer lymphedema, it may be supplemented with rehabilitation exercises involving active muscle contraction and relaxation alongside MLD/CDT [44]. Additionally, enhancing muscular strength and joint mobility, initiating early specialized training for various functional impairments [45], and employing physical therapy or surgical intervention when warranted can optimize limb functionality and elevate the quality of life for patients.

#### The volume and circumstance of the affected limb

A comparative examination of limb volume and circumference before and after treatment [21–27] demonstrated no distinct advantage of IPC in the treatment of breast cancer-related lymphedema, contradicting patient treatment outcome conclusions [28, 29]. The subjective evaluation criteria and insufficient methodological explanations in the included research cast doubt on the authenticity of the results. The role of IPC in lymphedema treatment is subtle, potentially attributable to chronic lymphedema, restricted action depth and range, or inadequate treatment planning and parameter configurations. Subgroup analyses indicated that IPC treatment was markedly more beneficial for patients with lymphedema lasting ≤ 24 months, implying superior efficacy in addressing freshly formed lymphedema relative to chronic instances. Furthermore, when IPC treatment pressure was ≤ 40 mmHg, a trend towards better therapeutic response was observed, suggesting that reduced pressure parameters may facilitate lymphatic return; it is hypothesized that lower pressure aligns more effectively with the physiological attributes of the lymphatic system, thereby promoting lymphatic flow and potentially contributing to modest improvement in limb volume, offering theoretical justification for clinical parameter configurations. In clinical applications, parameters including treatment pressure, duration, frequency, and pressure mode are frequently established based on clinical experience or established standards [46, 47]. Treatment durations exceeding 2 weeks appeared to yield more favorable outcomes, highlighting the need to develop optimized and standardized treatment strategies. In addition to efficacy considerations, the clinical application of IPC requires careful attention to safety and appropriate parameter selection, particularly regarding pressure intensity and treatment duration. These aspects are discussed in the following section.

### Safety profile and pressure optimization

This analysis confirmed the safety of IPC treatment, with no significant adverse responses, such as skin damage or exacerbation of local blood circulation abnormalities, observed in the included studies. Nonetheless, several possible security concerns remain to be acknowledged. A study indicated that lymphatic vessels sustained damaged when subjected to pressure ranging from 70 to 100 mmHg [47], and elderly individuals or those with skin disease may exhibit reduced tolerance to limb pressure. Retrospective investigations have suggested that sustained pressure should be maintained between 60 and 70 mmHg as the upper limit to avert damage [48]. A study indicated that IPC, when utilized as a secure supplementary treatment, is appropriate for home use in patients within the medium–low stress range [49]. Certain specialists concluded that when IPC is the principal treatment modality, optimal efficacy is achieved by setting the pressure between 30 and 60 mmHg and the treatment duration between 30 and 120 min [50].

### Controversial influence of IPC on subjective symptoms

The analysis of the included studies revealed that the impact of IPC devices on the alleviation of subjective symptoms in post-surgical lymphedema patients was controversial. Only one study indicated that BCRL patients utilizing IPC devices in conjunction with CDT treatment experienced alleviation of heaviness and tightness in the afflicted limb [22]. Conversely, the remaining four studies yielded negative results [21, 24–26]. The research identified variations in patient demographic features, intervention environments, and the sensitivity of evaluative instruments. Future endeavors must establish standardized criteria and conduct multicenter studies to precisely assess the genuine effect of IPC on patients’ subjective symptoms, hence informing enhancements in patient comfort and adherence.

## Limitations

First, language bias may exist as only Chinese and English studies were included. Second, heterogeneity in IPC protocols, including differences in pressure settings, session duration, treatment frequency, and device types, may have affected the validity of pooled estimates and limited the generalizability of the findings. Third, most preventive trials were single-center and small-sample studies conducted in China, which may limit the external validity and applicability of the preventive results to broader populations. Fourth, subjective symptom assessments lacked standardized tools, potentially affecting validity. A further limitation is the lack of blinding in outcome assessment across most included trials. As blinding is often challenging in rehabilitation studies, this may have increased detection bias and affected subjective measures such as limb volume or circumference. Multicenter RCTs with standardized IPC parameters, diverse populations, and long-term follow-up are needed to optimize protocols and confirm efficacy.

## Conclusions

This systematic review and meta-analysis evaluated the preventive and therapeutic effects of intermittent pneumatic compression (IPC) for breast cancer-related lymphedema. The findings suggest that IPC provides promising preventive benefits in reducing the incidence of lymphedema and limb dysfunction when applied early after breast cancer surgery. However, its therapeutic efficacy in established lymphedema remains inconsistent and uncertain, with benefits observed only under specific conditions (e.g., pressure ≤ 40 mmHg, treatment duration > 2 weeks). Given the methodological heterogeneity and limited evidence quality, further multicenter randomized controlled trials with standardized IPC protocols are required to confirm efficacy and guide clinical application.

## Registration and protocol

This systematic review and meta-analysis was conducted according to the Preferred Reporting Items for Systematic Review and Meta-Analysis (PRISMA) guidelines [51]. The study was registered at PROSPERO under the registration number CRD42025631301.

## Supplementary information

Below is the link to the electronic supplementary material.

ESM 1 (DOCX 132 KB)

## Data Availability

No datasets were generated or analysed during the current study.
