# Supplementary material for: Intermittent pneumatic compression devices for the prevention and treatment of breast cancer-related lymphedema—a systematic review and meta-analysis
Source: Support Care Cancer. 2025 Nov 22;33(12):1113. doi: 10.1007/s00520-025-10159-8 (PMC12638337; doi:10.1007/s00520-025-10159-8)
Supplement: Supplementary file 1 — (DOCX 132 KB) [file 520_2025_10159_MOESM1_ESM.docx]

**SUPPLEMENTARY DATA:**

Intermittent Pneumatic Compression Devices for the Prevention and Treatment of Breast Cancer-Related Lymphedema - A Systematic Review and Meta-analysis

Contents of Supplements

[Appendix 1 Inclusion and exclusion criteria and main outcomes for included studies 2](#_Toc119338440)

[Appendix 2 Bias risk of the included studies 5](#_Toc119338441)

[Appendix 3 Bias risk summary of the included studies 5](#_Toc119338442)

[Appendix 4 Evaluation of the methodological quality of literature 6](#_Toc119338443)

| Appendix 1. Inclusion and Exclusion Criteria and Main Outcomes for Included Studies | | | | |
| --- | --- | --- | --- | --- |
| Study | Inclusion Criteria | Exclusion Criteria | Reported Primary Outcome(s) | Main Results |
| Uzkeser et al^[27]^ | Patients with unilateral post-mastectomy lymphedema and no history of physical therapy with a difference of more than 2 cm in the circumference of a healthy limb or a difference of more than 10% in the volume of the two arms. | Patients who had bilateral lymphedema, current metastases, continuing radiotherapy, cellulites, venous thrombosis, elephantiasis, infection, lymphangiosis carcinomatosa, and congestive heart failure and those using any medications that affect the body fluid and electrolyte balance. | 1. Affected limb circumference /volume 2. Skin thickness 3. Pain level | There were significant differences in limb volume and circumference measurements between the experimental group and the control group before and after treatment, but no significant differences between the two groups. IPC also had no additional effect on patients' subjective perception. |
| Sanal-Toprak et al^[28]^ | Patients diagnosed with breast cancer who underwent breast surgery at least 3 months ago, aged 18 to 70 years, had stage 2 to 3 edema. | Patients with cellulitis, lymphangitis, fungal infections, lymph node metastases, and uncontrolled signs of psychosis or systemic disease. | 1. Affected limb circumference 2. Pain/tightness/heaviness scores | The experimental group and the control group showed significant improvement in the five measurement levels of upper arm circumference at week 5 and month 3, with no obvious superiority between the two. Shoulder ROM, pain, tension, and heaviness improved in both groups. |
| Gurdal et al^[22]^ | Patients who had undergone grade i or ii axillary lymph node dissection with radiotherapy for 6 months and had lymphedema. | Patients with bilateral lymphedema with a difference of less than 2cm in circumference measurements. | 1. Affected limb volume 2. ASES questionnaire score | Both groups could reduce the volume of affected limbs and improve the score of quality of life. But there was no significant difference between the two ways of treatment. |
| Haghighat et al^[26]^ | Patients who have been treated for breast cancer (surgery, chemotherapy, or radiation) for more than 3 months and have lymphedema. | Patients who active malignancy, breast cancer recurrence, active infection, patients with bilateral disease or bilateral lymphedema, venous insufficiency, low physical activity and unable to perform daily tasks, or female athletes with hyperphysical activity, history of previous treatment for lymphedema, neuromuscular diseases and any absolute contrain dications for CDT. | 1. Affected limb volume 2. Pain/heaviness/paraesthesia scores | Both CDT alone and MCDT combined with IPC can significantly reduce the limb volume in patients with postmastectomy lymphedema, with CDT alone providing better results in both phases of treatment. Both treatment methods can significantly reduce symptoms such as pain, heaviness, and paresthesia. |
| Tastaban et al^[23]^ | Patients who underwent surgery for unilateral breast cancer and completed chemoradiotherapy developed breast cancer-related unilateral upper arm lymphedema with a circumference difference greater than 2 cm or volume difference greater than 10%. | Patients who surgical intervention due to bilateral breast cancer, primer lymphoedema, undergoing complex decongestive therapy or other interventions for lymphoedema within 12 months, truncal and/or breast oedema, continuing chemotherapy and/or radiotherapy, infection, current metastases, heart failure with arterial and/or venous occlusion. | (1) Affected limb volume  (2) Pain/paraesthesia level | After treatment,there was a significant improvement in limb volume in both groups.The PREV of the experimental group (54.6%) was better than that of the control group (49.6%), but the difference between the groups was not statistically significant;the scores for the parameters of heaviness and tightness in the experimental group were more effective than those in the control group. |
| Szolnoky et al^[25]^ | Women with unilateral arm lymphedema more than 12 months after breast cancer surgery or adjuvant therapy. | Patients who subjects with any sign of local recurrence or distant metastases or if they were within the obligatory treatment-free period of one year. | 1. Affected limb volume 2. Subjective symptom questionnaire scores | The combined use of MLD and IPC provides a synergistic enhancement of the effects of CDP in terms of limb volume reduction. Although both groups showed improvement in subjective symptom scores, there were no significant differences between the groups at any time point. |
| Szuba et al^[24]^ | Patients with breast cancer-related lymphedema who have been treated for breast cancer (surgery or radiation, or both) for 12 weeks. | Patients presented with bilateral lymphedema, breast cancer recurrence, clinically active or clinically obvious infection, or venous occlusion. | 1. Affected limb volume 2. Tissue elasticity 3. Range of motion of the affected limb | The experimental group can effectively reduce the average volume of the affected limb compared to the control group, with a statistically significant difference; Moreover, IPC is well tolerated and significantly lacks complications. |
| YuNan, H et al^[30]^ | Patients diagnosed as breast cancer with CT and MRI diagnostic evidence, ultrasound diagnosis indicated the presence of inflammatory infiltration and the formation of single or multiple pus cavities. | Patients with severe other cancer conditions, intolerance to surgical treatment, recent use of anticoagulants, or renal dysfunction. | (1) Effective rate of limb edema  (2) Recovery rate of affected limb  (3) Recovery time of affected limb | The total effective rate and the excellent and good rate of limb function rehabilitation in IPC group were significantly higher than those in control group . The recovery time of limb function and hospitalization time in IPC group were shorter than those in control group. |
| YongHong, L et al^[29]^ | Women aged 38 to 59 years who developed lymphedema after unilateral breast cancer surgery received the first cycle of chemotherapy. | Bilateral breast cancer-associated lymphedema. | Effective rate of limb edema | The treatment effect of the affected limb edema in the experimental group was significantly better than that in the control group. |
| Yi, C et al^[20]^ | Patients diagnosed with breast cancer after surgery, without serious respiratory and circulatory diseases, with normal peripherality and normal activity of the affected limb before surgery, and with clear awareness and informed consent. | The patient has metastatic breast cancer; Skin infection of affected limb; Lymphedema due to reasons other than surgery; The affected limb was accompanied by deep vein thrombosis or thyroid disease. | (1) Lymphedema incidence  (2) Function of affected limb | Patients who have undergone breast cancer surgery can effectively alleviate lymphedema of the affected limb and promote the recovery of limb function by using gradient pressure therapy. After treatment,the degree of lymphedema in the study group was significantly lower than that in the control group, and the range of motion of the shoulder joint was significantly greater than that in the control group. |
| Na, L et al^[21]^ | Patients who meet the characteristics of modified radical mastectomy, are > 20 years old, have good cognitive ability and reading comprehension ability, and are willing to cooperate. | Patients with combined mental disorder, cognitive impairment, or recent use of psychotropic drugs; Patients with distant metastasis of cancer cells or breast conserving surgery. | (1) Lymphedema incidence  (2) Function of affected limb  (3) Quality of life | IPC effectively reduced the incidence of lymphedema, and the difference was statistically significant compared with the control group . The motion of shoulder joint in IPC group was better than that in control group. The total quality of life score of the patients after IPC intervention was higher than that of the control group. |
| YuPing, W et al^[17]^ | Female patients aged 30 to 60 years who underwent modified radical mastectomy for axillary lymph dissection. | Not described. | (1) Psychological status  (2) Lymphedema incidence | The incidence of lymphedema and the degree of swelling in the experimental group were lower than those in the control group and the anxiety of the patients was also significantly lower than that in the control group. |
| Jie, P et al^[18]^ | Women who pathological diagnosis of breast cancer, age ≥18 years and good coordination. | Patients with other tumors, abnormal liver and kidney function, and abnormal function of affected limb before surgery. | (1) Functional of affected limb  (2) Lymphedema incidence | IPC can significantly reduce the incidence and severity of lymphedema, but has no significant effect on shoulder motion. The incidence of lymphedema in the experimental group was lower than that in the control group. |
| Pinkun, L et al^[19]^ | The patient was diagnosed with breast cancer by pathological examination and modified radical resection, with stable condition and clear consciousness. | Patients with severe cardiovascular disease and limb dysfunction. | (1) Lymphedema incidence  (2) Affected limb circumference | The incidence of lymphedema on the affected upper limb of the observation group was lower than that of the control group; The difference of circumference diameter before and after treatment was significantly lower in the observation group than in the control group. |

Appendix 2. Bias risk of the included studies


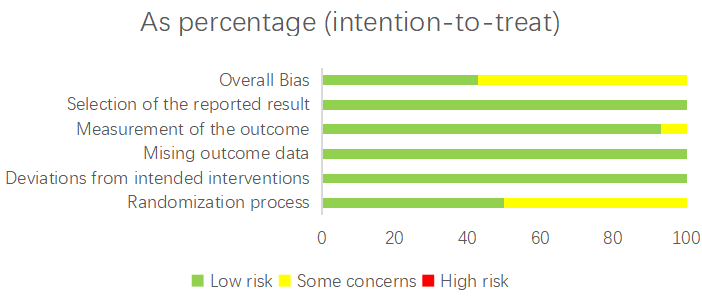


Appendix 3. Bias risk summary of the included studies


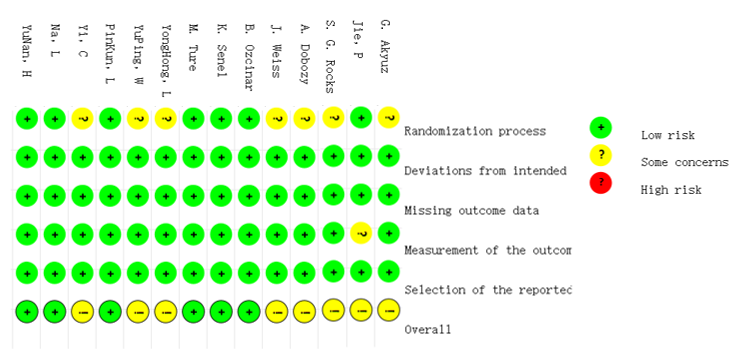


Appendix 4. Evaluation of the methodological quality of literature

| Study | Randomization process | Deviations from intended | Missing outcome data | Measurement of the outcome | Selection of the reported results | Level of evidence |
| --- | --- | --- | --- | --- | --- | --- |
| Uzkeser et al^[27]^ | low | low | low | low | low | low |
| Sanal-Toprak et al^[28]^ | low | low | low | low | low | low |
| Gurdal et al^[22]^ | low | low | low | low | low | low |
| Haghighat et al^[26]^ | Some concerns | low | low | low | low | Some concerns |
| Tastaban et al^[23]^ | low | low | low | low | low | low |
| Szolnoky et al^[25]^ | low | low | low | low | low | low |
| Szuba et al^[24]^ | Some concerns | low | low | low | low | Some concerns |
| YuNan, H et al^[30]^ | low | low | low | low | low | low |
| YongHong, L et al^[29]^ | Some concerns | low | low | low | low | Some concerns |
| Yi, C et al^[20]^ | Some concerns | low | low | low | low | Some concerns |
| Na, L et al^[21]^ | low | low | low | low | low | low |
| YuPing, W et al^[17]^ | Some concerns | low | low | low | low | Some concerns |
| Jie, P et al^[18]^ | low | low | low | Some concerns | low | Some concerns |
| Pinkun, L et al^[19]^ | low | low | low | low | low | low |
